# Comparative Metabolites and Citrate-Degrading Enzymes Activities in Citrus Fruits Reveal the Role of Balance between ACL and Cyt-ACO in Metabolite Conversions

**DOI:** 10.3390/plants9030350

**Published:** 2020-03-10

**Authors:** Lingxia Guo, Yongzhong Liu, Lijuan Luo, Syed Bilal Hussain, Yingxin Bai, Shariq Mahmood Alam

**Affiliations:** Key Laboratory of Horticultural Plant Biology (Ministry of Education), College of Horticulture and Forestry, Huazhong Agricultural University, Wuhan 430070, China; guolingxia@webmail.hzau.edu.cn (L.G.); Luolijuan@webmail.hzau.edu.cn (L.L.); bilal.hussain124@yahoo.com (S.B.H.); baiyingxin@webmail.hzau.edu.cn (Y.B.); m.smahmoodisb@yahoo.com (S.M.A.)

**Keywords:** ATP-citrate lyase, acetyl-CoA, citrus, fruit flesh quality, aconitase, metabolite conversion

## Abstract

Citric acid metabolism is considered to be the central cellular process of metabolite conversions. ATP-citrate lyase (ACL) and cytosolic aconitase (cyt-ACO) are the two citrate-degrading enzymes that decide the carbon flux towards different metabolite biosynthesis pathways. However, the correlation of their activities with metabolite concentrations in citrus fruits is still unclear. Here, the concentrations of soluble sugars, organic acids, acetyl-CoA, flavonoids, carotenoids, and γ-aminobutyric acid, as well as the activities of ACL, cyt-ACO, acetyl-CoA C-acetyltransferase, and acetyl-CoA carboxylase, were compared among the fruits of six citrus cultivars during fruit development and ripening. The results showed that the correlation between citrate concentration and cyt-ACO or ACL activity varied greatly among cultivars, while the activities of cyt-ACO and ACL had a significantly negative correlation (r = −0.4431). Moreover, ACL overexpression and RNA interference in the Citrus callus indicated that increasing and decreasing the ACL activity could reduce and induce cyt-ACO activity, respectively. In addition, significant correlation was only observed between the ACL activity and the concentration of acetyl-CoA (r = 0.4333). Taken together, the present study suggested that ACL and cyt-ACO synergistically control the citrate fate for the biosynthesis of other metabolites, but they are not the key determinants for the accumulation of citrate, as well as other metabolites in citrus fruits.

## 1. Introduction

Citrus fruit is a part of the human essential diet and is popularly consumed for its delicious taste and health promotion [1]. The edible part of citrus fruit contains low protein and fat, but abundant carbohydrates (sucrose, glucose, and fructose), organic acids (citric acid and malic acid), and some secondary metabolites, such as bioactive compounds (vitamins, carotenoids, flavonoids, limonoids) and aroma volatiles [2]. The combination of these metabolites in the cell vacuole or other organelles determines the fruit flesh quality [3,4,5,6].

**Figure 1:**
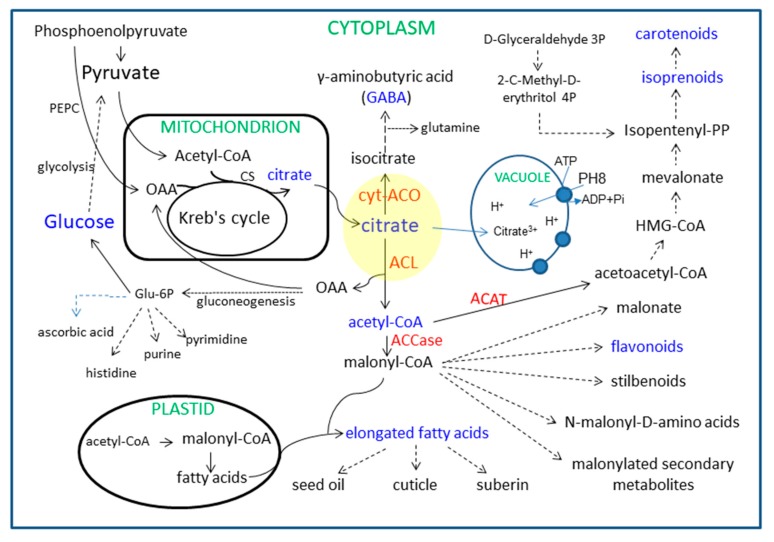
Schematic illustration of the network for metabolite connection in the plant cell. Yellow circle protrudes the crossroads of several pathways. Red letters indicate the key enzymes in the metabolite transformation and blue letters show the main metabolites. ACL refers to ATP-citrate lyase. cyt-ACO refers to cytosolic aconitase. ACAT refers to acetyl-CoA *C*-acetyltransferase. ACCase refers to acetyl-CoA carboxylase. PEPC refers to phosphoenolpyruvate carboxylase. CS refers to citrate synthase. PH8 refers to a P-type proton pump, *CsPH8* [18].

Fruit flesh quality is formed along with fruit development, which is coordinated with the conversion of phytochemicals, for example, starch to sugars, sugars to organic acids, and organic acids to amino acids or to some secondary metabolites [6,7]. These phytochemical conversions constitute a complex network that is closely associated via carbon flow (Figure 1). In brief, carbohydrates, such as glucose, are catabolized through the glycolysis into pyruvate, which actively transports into the mitochondrion and enters into the Kreb’s cycle accompanied by the input of acetyl-CoA; therein, citrate is synthesized by the condensation of oxaloacetate (OAA) and acetyl-CoA. Citrate can be oxidized in the Kreb’s cycle, or transported to the cytosol when the mitochondrial aconitase (ACO, EC 4.2.1.3) is partially blocked [8,9]. In the cytosol, except for storage in the vacuole, citrate can be directly catalyzed only by two key enzymes: cytosolic aconitase (cyt-ACO) and ATP-citrate lyase (ACL, EC 4.1.3.8). In the cyt-ACO catalyzing pathway, citrate is used for the biosynthesis of γ-aminobutyric acid (GABA) and amino acids [10,11], which contributes to the C: N balance [12]. In the ACL catalyzing pathway, citrate is cleaved into OAA and acetyl-CoA [13]. OAA can reenter into the mitochondrion for citrate biosynthesis or be used for gluconeogenesis or amino acid biosynthesis [14], while the cytosolic acetyl-CoA participates in the synthesis of many secondary metabolites through the catalysis of acetyl-CoA C-acetyltransferase (ACAT, EC 2.3.1.9) or acetyl-CoA carboxylase (ACCase, EC 6.4.1.2) [13,15,16,17]. 

As summarized in Figure 1, the cytosolic citrate utilization by the catalysis of cyt-ACO and ACL intersects with many metabolic pathways or conversions; moreover, cyt-ACO and ACL are in the center of metabolite conversion from soluble sugars to amino acids, fatty acids, flavonoids, and carotenoids. Especially, the resulting acetyl-CoA is the key node in these conversions. However, the acetyl-CoA is membrane impermeable and the flux of cytosolic acetyl-CoA only derives from the ACL-cleaved citrate degradation pathway [13,19], which cannot be compensated by other sources of acetyl-CoA [17]. Thus, the ACL function is pivotal in the regulation of glucose metabolism, citrate metabolism, fatty acid synthesis pathway, mevalonate pathway, and acetylation reactions [16,18]. 

Citrus is one of the world’s major fruit crops with global availability and popularity [20]. It is grown all over the world in more than 140 countries, and its global production is over 146 million metric tons in 2017 (FAO 2019). In China, the most well-known citrus fruits with commercial importance are orange, mandarin, and pumelo, of which the concentration and composition of metabolites in fruits are significantly varied [21]. Although the mechanism(s) for the accumulation of each metabolite, such as sugar, citrate, and antioxidants, has been studied for several decades [18,22,23], the contribution of metabolic interrelation to metabolite variations among different cultivars is unclear. This study compared the activities of citrate-degrading enzymes, as well as the concentrations of sugars, organic acids, flavonoids, carotenoids, and GABA, during fruit development of six citrus cultivars due to the significance of the cytosolic citrate utilization in metabolite conversions, and found that the balance of ACL and cyt-ACO activities plays a key role in deciding the citrate fate for metabolite conversions, but are not the determinants for the accumulation of related metabolites.

## 2. Results 

### 2.1. Changes in Soluble Sugar Concentration during Fruit Development and Ripening

Sucrose, glucose, and fructose are the main soluble sugars in citrus fruit juice sacs [2]. In the juice sacs of six cultivars, their accumulating process during fruit development and their final concentrations exhibited a big difference (Table 1). In each cultivar, the sucrose concentration was continuously and significantly increased during fruit development and ripening. At the T4 stage, the sucrose concentration was more than 60 mg·g^−1^ (FW) in ‘Newhall’ navel orange, ‘Guoqing No.1′ Satsuma mandarin and ‘Zaoxiang’ pumelo, while it was between 45 mg·g^−1^ (FW) and 50 mg·g^−1^ (FW) in ‘Anliu’ orange and ‘HB’ pumelo. ‘Huagan No. 2′ ponkan contained the lowest sucrose concentration [40.4 ± 1.85 mg·g^−1^ (FW)] among these cultivars. Interestingly, the concentrations of glucose and fructose had a similar change during the fruit development and ripening of each cultivar. In ‘Anliu’ orange, ‘Newhall’ navel orange, and ‘Guoqing No. 1′ Satsuma mandarin, the concentrations of glucose and fructose were continuously increased to the T4 stage. The final concentration of glucose was 39.9 mg·g^−1^ (FW), 41.8 mg·g^−1^ (FW), and 35.6 mg·g^−1^ (FW) in ‘Anliu’ orange, ‘Newhall’ navel orange, and ‘Guoqing No. 1′ Satsuma mandarin, respectively, while the final concentration of fructose in these cultivars was 45.9 mg·g^−1^ (FW), 50.4 mg·g^−1^ (FW), and 44.5 mg·g^−1^ (FW), respectively. In ‘Huagan No.2′ ponkan, the concentrations of glucose and fructose were slightly reduced during fruit development and ripening. The concentrations of glucose and fructose were 4.7 mg·g^−1^ (FW) and 3.6 mg·g^−1^ (FW), respectively, at the T4 stage (205 days after anthesis (DAA)). In ‘Zaoxiang’ pumelo, the concentrations of glucose and fructose obviously fluctuated during fruit development and ripening, and their final concentrations were 18.2 mg·g^−1^ (FW) and 21.9 mg·g^−1^ (FW), respectively. In ‘HB’ pumelo, the concentrations of glucose and fructose were significantly decreased from 86 DAA (T1) to 115 DAA (T2) and then significantly increased to 195 DAA (T4) when their concentrations were 38.9 mg·g^−1^ (FW) and 40.2 mg·g^−1^ (FW), respectively.

### 2.2. Changes in Organic Acids Concentration during Fruit Development and Ripening

Citric acid and malic acid are the two main organic acids in citrus fruit juice sacs [2], but quinic acid is the major organic acid during early fruit development [24]. Therefore, this study comparatively analyzed the three organic acids in the juice sacs of six citrus cultivars (Table 2). Except for ‘Huagan No. 2′ ponkan, of which the citrate concentration was continuously and significantly decreased during fruit development and ripening, the citrate concentration in the other five cultivars had a similar profile, which was increased at the early stages and then decreased during ripening. However, the final citrate concentration varied among the six cultivars. It was 6.6 mg·g^−1^ (FW), 9.1 mg·g^−1^ (FW), 9.1 mg·g^−1^ (FW), 5.0 mg·g^−1^ (FW), 5.7 mg·g^−1^ (FW), and 9.4 mg·g^−1^ (FW) in ‘Anliu’ orange, ‘Newhall’ navel orange, ‘Guoqing No. 1′ Satsuma mandarin, ‘Huagan No. 2′ ponkan, ‘Zaoxiang’ pumelo, and ‘HB’ pumelo, respectively. On the other hand, the concentrations of malic acid and quinic acid in all of the cultivars kept a decreasing trend during fruit development and ripening. At the T4 stage, their concentrations were less than 1.0 mg·g^−1^ (FW), except for ‘Anliu’ orange and ‘Guoqing No. 1′ Satsuma mandarin, of which the malic acid concentration was 1.1 mg·g^−1^ (FW) and 1.2 mg·g^−1^ (FW), respectively.

### 2.3. Changes in Cytosolic Aconitase (cyt-ACO) Activity and γ-Aminobutyric Acid (GABA) Concentration during Fruit Development and Ripening

**Figure 2:**
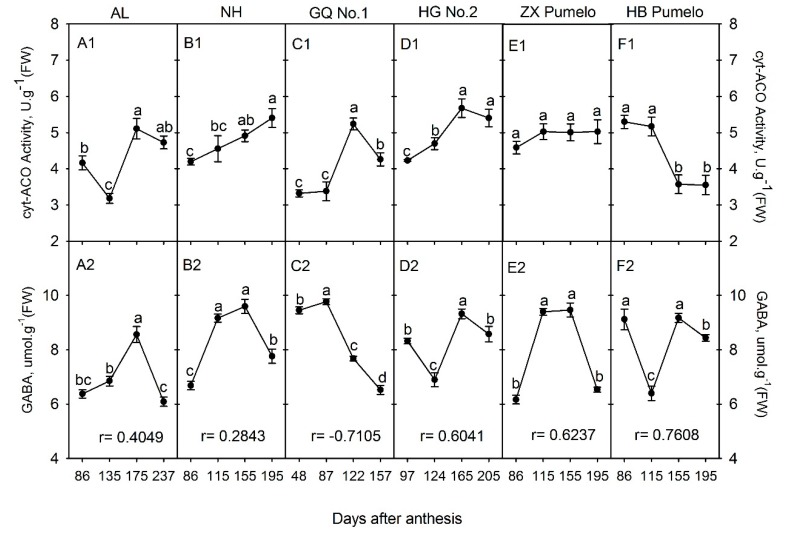
Changes of the cytosolic aconitase (cyt-ACO) activity (**A1~F1**) and γ-aminobutyric acid (GABA) concentration (**A2~F2**) in the fruit juice sacs during fruit development and ripening of six citrus cultivars. AL refers to ‘Anliu’ orange. NH refers to ‘Newhall’ navel orange. GQ No. 1 refers to ‘Guoqing No. 1′ Satsuma mandarin. HG No.2 refers to ‘Huagan No. 2′ ponkan. ZX pumelo refers to ‘Zaoxiang’ pumelo. HB pumelo refers to ‘HB’ pumelo. Different lowercase letters on the bar in each graph indicate significant differences at *p* < 0.05 among samples at different stages by Duncan’s multiple range test. r refers to the correlation coefficient between the GABA concentration and the cyt-ACO activity in each cultivar.

The citrate can be directly catalyzed by cyt-ACO to form iso-citrate, subsequently converted to α-ketoglutarate, which can enter into the GABA-shunt pathway for the biosynthesis of GABA [10,25]. In this study, the cyt-ACO activity and GABA concentrations were compared in the juice sacs of six citrus cultivars during fruit development. It was clearly found that the changes in cyt-ACO activity and GABA concentration varied among cultivars (Figure 2). The cyt-ACO activity in ‘Anliu’ orange was significantly decreased from 86 DAA to 135 DAA, and then significantly increased to the peak at 175 DAA and slightly decreased again at 237 DAA (Figure 2A1). In ‘Newhall’ navel orange (Figure 2B1), its activity was continuously, but significantly, increased from 86 DAA to 195 DAA. The profile of cyt-ACO activity in ‘Guoqing No. 1′ Satsuma mandarin was similar to ‘Huagan No. 2′ ponkan, which was significantly increased to the peak at 122 DAA in ‘Guoqing No. 1′ Satsuma mandarin or at 165 DAA in ‘Huagan No. 2′ ponkan, and then decreased significantly or slightly (Figure 2C1,D1). Differently, the cyt-ACO activity in ‘Zaoxiang’ pumelo kept an almost constant level from 86 DAA to 195 DAA (Figure 2E1), while its activity was significantly decreased from 86 DAA to 195 DAA in ‘HB’ pumelo (Figure 2F1).

As for the GABA, its concentration in ‘Anliu’ orange was significantly increased to the peak at 175 DAA, and then significantly decreased at 237 DAA (Figure 2A2). In ‘Newhall’ navel orange (Figure 2B1) and ‘Zaoxiang’ pumelo (Figure 2E2), the change of GABA concentration had a similar profile, which was significantly increased from 86 DAA to 115 DAA, followed by a constant level from 115 DAA to 155 DAA, and then significantly decreased at 195 DAA. In ‘Guoqing No. 1′ Satsuma mandarin, the GABA concentration was significantly increased to the peak at 87 DAA and then significantly decreased to the lowest level, at 157 DAA (Figure 2C2). Moreover, the change of GABA concentration also had a similar profile in ‘Huagan No. 2′ ponkan (Figure 2D1) and ‘HB’ pumelo (Figure 2F1), which fluctuated significantly during fruit development and ripening. Moreover, the correlation of GABA concentration with cyt-ACO activity was positive in all cultivars, except ‘Guoqing No. 1′ Satsuma mandarin, which had a negative correlation coefficient (Figure 2). 

### 2.4. Changes in ACL Activity and Acetyl-CoA Concentration during Fruit Development and Ripening

**Figure 3:**
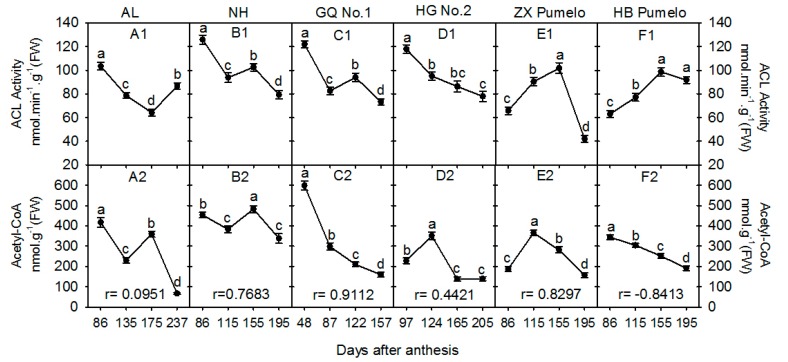
Changes of ATP-citrate lyase (ACL) activity (**A1~F1**) and acetyl-CoA concentration (**A2~F2**) in the fruit juice sacs during fruit development and ripening of six citrus cultivars. AL refers to ‘Anliu’ orange. NH refers to ‘Newhall’ navel orange. GQ No. 1 refers to ‘Guoqing No. 1′ Satsuma mandarin. HG No. 2 refers to ‘Huagan No. 2′ ponkan. ZX pumelo refers to ‘Zaoxiang’ pumelo. HB pumelo refers to ‘HB’ pumelo. Different lowercase letters on the bar in each graph indicate significant differences at *p* < 0.05 among samples at different stages by Duncan’s multiple range test. r refers to the correlation coefficient between the acetyl-CoA concentration and the ACL activity in each cultivar.

The ACL is another cytosolic enzyme that catalyzes the citrate into acetyl-CoA [16,26]. Here, the changes in ACL activity and acetyl-CoA concentration also varied among six cultivars during fruit development (Figure 3). The ACL activity in ‘Anliu’ orange was significantly decreased from 86 DAA to 175 DAA, and then significantly increased at 237 DAA (Figure 3A1). In ‘Newhall’ navel orange (Figure 3B1), ‘Guoqing No. 1′ Satsuma mandarin (Figure 3C1), and ‘Huagan No. 2′ ponkan (Figure 3D1), their ACL activities showed a significant decrease during fruit development. In addition, the ACL activities in two pumelo cultivars showed a similar changing pattern, which was significantly increased from 86 DAA to 155 DAA, and then was significantly or slightly decreased at 195 DAA in ‘Zaoxiang’ or ‘HB’ pumelo, respectively (Figure 3E1 or Figure 3F1). 

As for the acetyl-CoA, its concentration showed a decreasing trend with significant fluctuation in both ‘Anliu’ orange (Figure 3B1) and ‘Newhall’ navel orange (Figure 3B2). However, in the last stage, its concentration was 66.6 nmol·g^−1^ (FW) in ‘Anliu’ orange (Figure 3B1) and 337.9 nmol·g^−1^ (FW) in ‘Newhall’ navel orange (Figure 3B2). In ‘Guoqing No. 1′ Satsuma mandarin, the acetyl-CoA concentration at 48 DAA was the maximum [598.4 nmol·g^−1^ (FW)], and then significantly decreased to 159.3 nmol·g^−1^ (FW) at 157 DAA (Figure 3C2). In ‘Huagan No.2′ ponkan (Figure 3D2) and ‘Zaoxiang’ pumelo (Figure 3E2), the acetyl-CoA concentration showed a similar pattern, which was significantly increased during early fruit development, and then significantly decreased during the later fruit development; in the last stage, its concentration was 139.0 nmol·g^−1^ (FW) in ‘Huagan No. 2′ ponkan (Figure 3D2), and 156.8 nmol·g^−1^ (FW) in ‘Zaoxiang’ pumelo (Figure 3E2). The acetyl-CoA concentration in ‘HB’ pumelo also showed a significant decrease during fruit development, similar to ‘Guoqing No. 1′ Satsuma mandarin; however, its concentration was 344.2 nmol·g^−1^ (FW) at 86 DAA and 190.9 nmol·g^−1^ (FW) at 195 DAA (Figure 3F2). Moreover, a very low positive correlation of acetyl-CoA concentration with ACL activity was found in ‘Anliu’ orange (r = 0.0951) and a higher positive correlation was found in ‘Newhall’ navel orange (r = 0.7683), ‘Guoqing No. 1′ Satsuma mandarin (r = 0.9112), ‘Huagan No. 2′ ponkan (r = 0.4421) and ‘Zaoxiang’ pumelo (r = 0.8297); while, the correlation was −0.8413 in ‘HB’ pumelo (Figure 3).

### 2.5. Changes in Acetyl-CoA C-Acetyltransferase (ACAT) and Carotenoids Concentration during Fruit Development and Ripening

**Figure 4:**
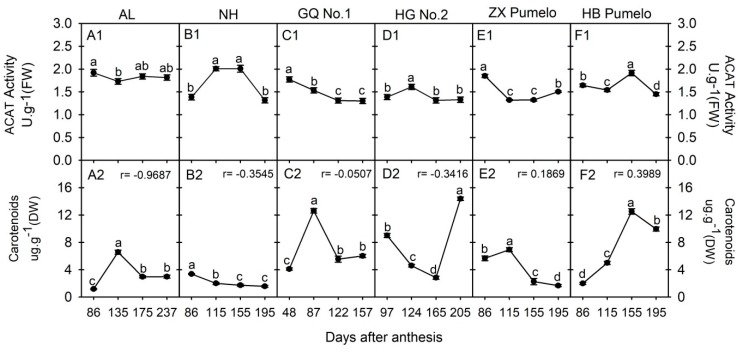
Changes of acetyl-CoA C-acetyltransferase (ACAT) activity (**A1~F1**) and carotenoids concentration (**A2~F2**) in the fruit juice sacs during fruit development and ripening of six citrus cultivars. AL refers to ‘Anliu’ orange. NH refers to ‘Newhall’ navel orange. GQ No. 1 refers to ‘Guoqing No. 1′ Satsuma mandarin. HG No. 2 refers to ‘Huagan No. 2′ ponkan. ZX pumelo refers to ‘Zaoxiang’ pumelo. HB pumelo refers to ‘HB’ pumelo. Different lowercase letters on the bar in each graph indicate significant differences at *p* < 0.05 among samples at different stages by Duncan’s multiple range test. r refers to the correlation coefficient between the carotenoid concentration and the ACTA activity in each cultivar.

The cytosol acetyl-CoA can enter into the mevalonate pathway through the catalysis of ACAT, which is the first enzyme that converts the acetyl-CoA into acetoacetyl-CoA; on the other hand, carotenoids are terminal products of the mevalonate pathway (Figure 1). The ACAT activity and total carotenoids concentration were comparatively analyzed in the juice sacs of six citrus cultivars during fruit development (Figure 4). In ‘Anliu’ orange, the ACAT activity was significantly decreased from 86 DAA to 135 DAA, and then slightly fluctuated during the following stages (Figure 4A1). In ‘Newhall’ navel orange, the ACAT activity was significantly increased from 86 DAA to 115 DAA, peaked at both 115 DAA and 155 DAA, and then significantly decreased at 195 DAA (Figure 4B1). In ‘Guoqing No. 1′ Satsuma mandarin, the ACAT activity showed a significantly decreased trend during fruit development (Figure 4C1), and in ‘Huagan No. 2′ ponkan, it was significantly increased from 97 DAA to 124 DAA, and then significantly declined to the similar level at 97 DAA (Figure 4D1). Contrary to ‘Newhall’ navel orange, the ACAT activity in ‘Zaoxiang’ pumelo was significantly decreased from 86 DAA to 115 DAA, and then kept a similar level from 115 DAA to 155 DAA, followed by a significant increase at 195 DAA (Figure 4E1). In ‘HB’ pumelo, the ACAT activity showed a significant fluctuation during fruit development and ripening (Figure 4F1).

As for the total carotenoids, its concentration in the juice sacs of ‘Anliu’ orange was significantly increased, peaked at 135 DAA, and then significantly decreased to 237 DAA (Figure 4A2). In ‘Newhall’ navel orange, the carotenoid concentration was significantly decreased during fruit development (Figure 4B2). In ‘Guoqing No. 1′ Satsuma mandarin, the carotenoids concentration had a similar changing profile as in ‘Anliu’ orange, which was significantly increased during the first two sampling points, and then significantly decreased and kept a similar level at the last two sampling points (Figure 4C2). In ‘Huagan No. 2′ ponkan, the carotenoids concentration was significantly decreased from 97 DAA to 165 DAA, and then significantly increased to the highest level at 205 DAA (Figure 4D2). The carotenoids concentration in ‘Zaoxiang’ pumelo was significantly decreased during the first two sampling points, similar to the ‘Anliu’ orange and ‘Guoqing No. 1′ Satsuma mandarin; however, it then was significantly and continuously declined to the last sampling point (Figure 4E2). Contrary to ‘Huagan No. 2′ ponkan, the carotenoids concentration in ‘HB’ pumelo was significantly increased during the first three sampling points, and then significantly decreased to the last sampling point (Figure 4F2). In addition, the correlation coefficient between the total carotenoids concentration and ACAT activity was negative in orange or tangerine cultivars, but it had a positive correlation in pumelo cultivars (Figure 4).

### 2.6. Changes in Acetyl-CoA Carboxylase Activity (ACCase), Malonyl-CoA and Flavonoids Concentration during Fruit Development and Ripening

**Figure 5:**
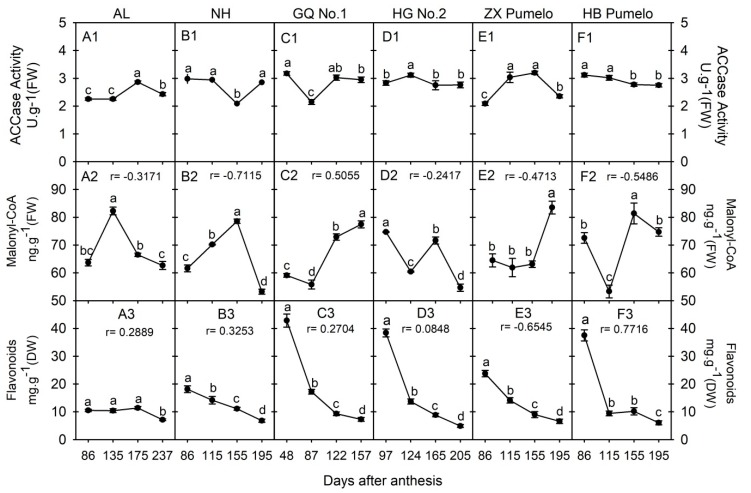
Changes of acetyl-CoA carboxylase (ACCase) activity (**A1~F1**), malonyl-CoA (**A2~F2**) and flavonoids (A3~F3) concentration in the fruit juice sacs during fruit development and ripening of six citrus cultivars. AL refers to ‘Anliu’ orange. NH refers to ‘Newhall’ navel orange. GQ No. 1 refers to ‘Guoqing No. 1′ Satsuma mandarin. HG No. 2 refers to ‘Huagan No. 2′ ponkan. ZX pumelo refers to ‘Zaoxiang’ pumelo. HB pumelo refers to ‘HB’ pumelo. Different lowercase letters on the bar in each graph indicate significant differences at *p* < 0.05 among samples at different stages by Duncan’s multiple range test. r refers to the correlation coefficient between the malonyl-CoA or flavonoids concentration and the ACCase activity in each cultivar.

ACCase has the function of catalyzing the irreversible carboxylation of acetyl-CoA to produce malonyl-CoA, which is the substrate for the biosynthesis of flavonoids (Figure 1). Here, we comparatively analyzed the ACCase activity, malonyl-CoA, and flavonoids concentration in the juice sacs of six citrus cultivars during fruit development (Figure 5). The ACCase activity in ‘Anliu’ orange was significantly increased from 135 DAA to 175 DAA, and then significantly decreased at 237 DAA (Figure 5A1). In ‘Newhall’ navel orange, the ACCase activity was significantly decreased from 115 DAA to 155 DAA, and then significantly increased to the same level as it was at the first two sampling points (Figure 5B1). The ACCase activity profile in ‘Guoqing No.1′ Satsuma mandarin was similar to that in ‘Newhall’ navel orange; however, the lowest level appeared at the second sampling point (Figure 5C1). In ‘Huagan No. 2′ ponkan, the ACCase activity showed a similar changing profile to that in ‘Anliu’ orange, but the highest level appeared at the second sampling point (Figure 5D1). In ‘Zaoxiang’ pumelo, the ACCase activity was significantly increased to the peak at 155 DAA, and then significantly decreased at 195 DAA (Figure 5E1). In ‘HB’ pumelo, the ACCase activity was the same at the first two sampling points, followed by a significant decrease at the third sampling point, and then was kept a constant level to the fourth sampling point (Figure 5F1). 

As for the malonyl-CoA, its concentration in ‘Anliu’ orange was significantly increased to peak at 135 DAA, and then significantly decreased to 237 DAA (Figure 5A2). In ‘Newhall’ navel orange, the malonyl-CoA concentration was significantly increased and reached a peak at 155 DAA, and then significantly decreased to 195 DAA (Figure 5B2). In ‘Guoqing No. 1′ Satsuma mandarin, the malonyl-CoA concentration was significantly decreased to 87 DAA, and then significantly increased to peak at 157 DAA (Figure 5C2). The malonyl-CoA concentration in ‘Huagan No. 2′ ponkan (Figure 5D2) had a similar decrease-increase-decrease profile to that in ‘HB’ pumelo (Figure 5F2). In ‘Zaoxiang’ pumelo, the malonyl-CoA concentration kept a constant level from 86 DAA to 155 DAA, and then significantly increased at 195 DAA (Figure 5E2). Moreover, the correlation coefficient between malonyl-CoA concentration and ACCase activity was negative in all cultivars, except for ‘Guoqing No. 1′, which had a positive correlation (Figure 5A2~F2). 

As for the total flavonoids, its concentration in ‘Anliu’ orange varied a little during fruit development; only the concentration at 237 DAA was significantly lower than the other sampling points (Figure 5A3). Moreover, the changing profiles of the total flavonoids’ concentration were similar in the other five cultivars; namely, they significantly decreased during fruit development and the lowest level appeared at the last sampling point (Figure 5B3~F3). In addition, the correlation coefficient between the total flavonoids concentration and ACCase activity was positive in all cultivars, except for ‘Zaoxiang’ pumelo, which had a negative correlation (as in Figure 5A3~F3).

### 2.7. Change in ACL Activity Could Alter the Cyt-ACO Activity as well as Citrate, Acetyl-CoA, GABA, Total Flavonoids, and Carotenoids Concentrations

**Figure 6:**
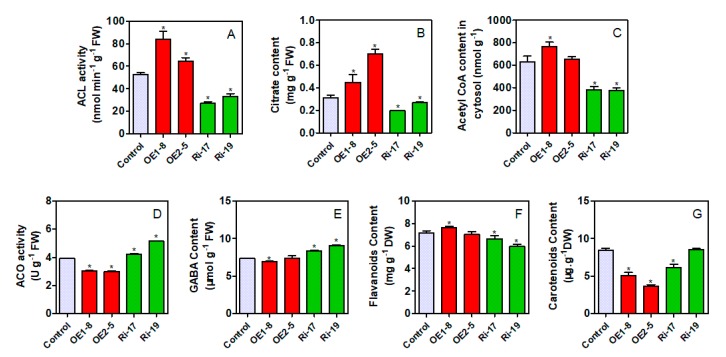
Changes of ACL activity (**A**), citrate concentration (**B**), acetyl-CoA concentration (**C**), cyt-ACO activity (**D**), GABA (**E**), flavonoids (**F**), and carotenoids (**G**) concentrations in citrus callus lines of *CitACLα1β1* overexpression (OE) and RNA interference (Ri). Asterisk (*) indicates that the difference is significant between the transgenic line and the control at *p* < 0.05 (*t*-test).

Two citrus callus lines of ACL overexpression (OE) and ACL RNA interference (Ri) were selected for further analysis. The ACL activities in both OE lines were significantly increased as compared to the control, while significantly decreased in both Ri lines (Figure 6A). Correspondingly, the citrate concentrations were significantly increased in both OE lines and significantly decreased in both Ri lines (Figure 6B); the acetyl-CoA concentration(s) was significantly increased in OE1–8 and significantly decreased in both Ri lines (Figure 6C). Moreover, the activities of cyt-ACO and the concentrations of GABA were altered either in the transgenic lines. The activities of cyt-ACO were significantly decreased in both OE lines and were significantly increased in both Ri lines (Figure 6D). As for the GABA, its concentration was significantly decreased in OE1–8, while it was significantly increased in both Ri lines as compared to the control (Figure 6E). In addition, the total flavonoids and carotenoids were also detected in the transgenic lines. The total flavonoids concentration was only significantly higher in OE1–8, while was significantly lower in both Ri lines than that in the control (Figure 6F); as for the total carotenoids, its concentration was significantly lower in either OE lines or Ri-17 (Figure 6G).

## 3. Discussion

Citrus fruits are highly attractive to consumers because of their richness in health-related and sensorial attributes [1]. These beneficial attributes constitute an important part of fruit internal quality and they are mainly decided by the metabolites in fruit pulp (edible part). Metabolites in the fruit pulp are synthesized during fruit development [18,23,27,28,29], and their concentrations largely vary among citrus species and varieties [21,24,30]. Of most citrus fruits, the fruit pulp contains low protein and fat, but abundant carbohydrates, organic acids, and secondary metabolites, such as carotenoids and flavonoids [2]. Hence, we here just compared the concentrations of soluble sugars, organic acids, carotenoids, and flavonoids among six citrus cultivars and found that the concentration of each metabolite had a big difference among the cultivars, similar to the previous results [21,24,30].

Metabolite biosynthesis or conversion is a complicated process in the plant cell. Investigating the key factor responsible for metabolite variations among cultivars becomes very attractive to scientists, because it conduces the improvement of fruit quality or health-beneficial components [1,31]. To date, the variation of some specific metabolites has been well studied. For example, organic acids are important components in determining the fruit organoleptic quality [22]. Citric acid is the predominant organic acid in citrus fruit (Table 2) and citrus fruit acidy is mainly formed by the accumulation of citric acid in the vacuole [18]. A recent review indicated that citrate-metabolizing enzymes, including citrate synthase, ACO, ACL, and proton pumps, are involved in the accumulation of citric acid; however, ACO and ACL are not the key factors deciding the diversity of citric acid among the fruits of different cultivars [18]. The present results showed that the correlation of citrate concentration and cyt-ACO or ACL varied greatly among the cultivars (Table 3); moreover, the correlation coefficient of ACL or cyt-ACO with citrate concentration without considering cultivars was 0.3946 or −0.3404 with no significance (Table S1). These results supported that cyt-ACO and ACL are involved in the citrate degradation, but are not the key factors in the determination of citrate concentration in the citrus fruits. In theory, the overexpression of *ACL* genes should decrease the citrate concentration and RNA interference of *ACL* genes should increase the citrate concentration. However, the current gene transformation in citrus callus resulted in a reverse phenomenon (Figure 6B). We further detected the expression levels of genes that were related to citrate biosynthesis (*CS1* and *PEPCs*) and transport (*PH8*) (Figure S1), and found that the expression of *CS1* was significantly increased in both OE lines, but significantly decreased in both Ri lines (Figure S1D); moreover, no significant difference was observed in the expression levels of *PEPCs* and *PH8* between the transgenic lines and the control (Figure S1). These results suggested that the overexpressing *ACL* significantly increased the citrate-synthesizing ability, but the RNA interfering *ACL* significantly decreased the citrate-synthesizing ability, which is possibly the reason for the reverse response of citrate to the alteration of ACL activity in transgenic callus. Moreover, these results further displayed the lesser role of ACL in the regulation of citrate concentration [32]. 

Although the accumulation of most phytochemicals in fruits involves metabolite biosynthesis, transport, degradation, and storage [18,22,23], the metabolism of each metabolite is not a separate event, but rather connects each other through carbon flow via organic acid metabolism (Figure 1) [14]. For example, Iglesias et al. [33] found that *in vitro* and *in vivo* accumulation of sucrose stimulated the color change from green to orange in mandarin peel at the ripening stage, while, in tomato, sucrose limitation reduced the lycopene and phytoene concentrations in pericarp discs of mature green fruits [34]. Figure 1 shows that the connection from sucrose to carotenoids involves sucrose metabolism, glycolysis, citric acid metabolism, and mevalonate pathway. Despite being involved in nutritional and hormonal signals [33,35], these metabolite interactions should be related to organic acid metabolism [14] and especially to the function of cyt-ACO and ACL [11,36,37]. The previous study indicated that even moderately reduced ACL activity will cause a complex, dwarf phenotype, with miniaturized organs, smaller cells, aberrant plastid morphology, and reduced cuticular wax deposition [17]. In this study, we found that the activities of cyt-ACO (Figure 2) and ACL (Figure 3) had almost a reverse changing trend during fruit development in the studied cultivars and they had a significant correlation coefficient of −0.4431 (Table S1). Namely, when the activity of cyt-ACO kept a decreasing trend during fruit development, for example, in ‘Newhall’ navel orange (Figure 2B1), the activity of ACL almost displayed an increasing trend (Figure 3B1), and vice versa. This balance status demonstrated that the activities of cyt-ACO and ACL synergistically control the citrate fate for the biosynthesis of different metabolites in citrus fruits juice sacs. Moreover, modulating ACL activity could alter the cyt-ACO activity, as well as the concentrations of citrate, acetyl-CoA, GABA, flavonoids, and carotenoids (Figure 6). The current results confirmed the strategic role of cyt-ACO and ACL in the metabolite conversion. Although the acetyl-CoA that is produced by the catalysis of ACL can enter into the flavonoids and carotenoids biosynthesis pathways (Figure 1) [17], it was interestingly found that the concentration of flavonoids, rather than carotenoids, was consistent with the change of ACL activity in transgenic callus (Figure 6). These results suggested that ACL has more influence on flavonoids concentration than carotenoids concentration.

When the cytosolic citrate is degraded by cyt-ACO, the carbon will flow into the GABA-shunt pathway for GABA biosynthesis [10,25]. Although the carbon skeletons for GABA biosynthesis derives from the citrate that is catalyzed by cyt-ACO, the activity of cyt-ACO is not the key enzyme for modulating the plant cell GABA concentration, which is decided by the balance of the glutamate concentration, glutamate decarboxylase, and GABA transaminase [38]. Here, the correlation coefficient between cyt-ACO and GABA concentration varied among the cultivars (Figure 2) and the correlation coefficient (not considering cultivars) was 0.1959 with no significance (Table S1), thus suggesting the lesser role of cyt-ACO in the regulation of GABA concentration. On the other hand, when the cytosolic citrate is degraded by ACL, the carbon will flow into the process for the biosynthesis of secondary metabolites or the biosynthesis of long-chain fatty acids through the production of acetyl-CoA (Figure 1). The cytosolic acetyl-CoA is membrane impermeable and only derives from the citrate that is catalyzed by ACL [13,19]. In the cytosol, the acetyl-CoA can be catalyzed by ACAT or ACCase for the biosynthesis of long-chain fatty acids or secondary metabolites [13,16]. It seems that ACL, ACAT, and ACCase should play important roles in regulating the acetyl- CoA concentration, and then the concentrations of long-chain fatty acids or secondary metabolites. The overexpression of ACL genes or reducing the ACL activity significantly influenced the cytosolic acetyl-CoA concentration and the accumulation of cytosolic acetyl-CoA-derived metabolites [15,17]. Current gene transformation also found that the overexpression of ACL genes or reduction of the ACL activity could significantly influence the cytosolic acetyl-CoA concentration (Figure 6C). However, the correlation between the acetyl-CoA concentration and the activity of ACL, ACAT, or ACCase varied among citrus cultivars (Figure 3 and Table S2), although the correlation (not considering cultivars) between the acetyl-CoA concentration and the ACL activity was significantly positive (Table S1). Moreover, there was no strong positive correlation between the ACCase activity and the flavonoids concentration or the ACAT activity and the carotenoids concentration (Table S1). These results suggested that the ACL and cyt-ACO are not the key factors in deciding the final concentration of a given metabolite, although the balance of ACL and cyt-ACO activities decides the citrate fate in the cell. In addition, the present results also found that neither overexpressing nor RNA interfering *ACL* significantly increased carotenoids concentration (Figure 6G), which suggested that the carotenoids biosynthesis is less subjected to the regulation of the mevalonate pathway, but possibly to the methylerythritol 4-phosphate pathway [39].

## 4. Materials and Methods

### 4.1. Plant Materials

The fruits of ‘Anliu’ orange (*Citrus sinensis* cv. Anliu), ‘Newhall’ navel orange (*C. sinensis* cv. Newhall), ‘Guoqing No.1′Satsuma mandarin (*C. unshiu* cv. Guoqing No. 1), ‘Huagan No. 2′ ponkan (*C. reticulata* cv. Huagan No. 2), ‘Zaoxiang’ pumelo (*C. grandis* cv. Zaoxiang), and ‘HB’ pumelo (*C. grandis* cv. HB) were collected at four developmental stages (T1, T2, T3, and T4) from the citrus germplasm orchard in the Huazhong Agricultural University (Wuhan, Hubei Province, China). In detail, the ‘Anliu’ fruits were harvested at 86, 135, 175, and 237 DAA. ‘Newhall’, ‘Zaoxiang’, and ‘HB’ pumelo fruits were harvested at 86, 115, 155, and 195 DAA. ‘Guoqing No. 1’ fruits were harvested at 48, 87, 122, and 157 DAA. ‘Huagan No. 2′ fruits were harvested at 97, 124, 165, and 205 DAA. The last sampling point in each cultivar was in the ripening stage. At each sampling point, five to ten healthy fruits were randomly harvested from the tree outer crown. The fruit juice sacs of each sample were separated and immediately ground into granules in liquid nitrogen (N_2_) and stored at −70 °C for further experiments.

### 4.2. Determination of Soluble Sugars, Organic Acids, γ-Aminobutyric Acid (GABA), Acetyl-CoA, Malonyl-CoA, Total Flavonoids, and Carotenoids

Frozen granules of each sample were mulled with liquid N_2_ into a fine powder. Approximately 3 g fine powder sample was used for the determination of glucose, fructose, sucrose, citric acid, malic acid, and quinic acid with gas chromatography, according to the method of Bartolozzi et al. [40]. The supernatant was filtered through a 0.22 μm filter and it was subjected to gas chromatography analysis using an Agilent 7890B gas chromatograph (Agilent, USA) with a flame ionization detector. A nonpolar HP-5 (5%-Phenyl-methyl polysiloxane, 30 m × 0.32 mm i.d. × 0.25 μm) column was used for separation. For γ-aminobutyric acid (GABA), approximately 0.1 g fine powder was used for the rapid determination of GABA by a spectrophotometric assay method, as described before [41]. For acetyl-CoA concentration, the mitochondria were separated using a Mitochondrial Isolation Kit (Suzhou Comin Biotechnology Co, Ltd., Suzhou, China) to collect cell disruption fluid and cytoplasm, and the acetyl-CoA concentration in the cytoplasm was then detected following the instructions of Acetyl-CoA Assay Kit (Suzhou Comin Biotechnology Co, Ltd., Suzhou, China). Approximately 0.1 g fine powder sample was used for the determination of the malonyl-CoA concentration and analyzed by Enzyme-Linked Immunosorbent Assay (ELISA) Kit (plant) (Jiangsu Meibiao Biological Technology Co., Ltd., Jiangsu, China).

For the total flavonoids and carotenoids examination, about 4.0 g of each sample was freeze-dried using a vacuum freeze dryer and then ground into powder. About 0.02 g freeze-dried sample was used for determinations, following the protocol of Plant Flavonoids Test Kit and Plant Carotenoids Test Kit (Suzhou Comin Biotechnology Co., Ltd., Suzhou, China), respectively. 

### 4.3. Determination of ATP-Citrate Lyase, Cyt-Aconitase, Acetyl-CoA Carboxylase, and Acetyl-CoA Acetyltransferase Activities

The ACL activity was determined using the method that was described by Langlade et al. [42], with little modification. About 1.0 g fine powder sample was homogenized with 3 mL extraction buffer (0.1 M KH_2_PO_4_, 50 mM NaF, 0.1 mM EDTA, 1 mM MgCl_2_, and 1 mM DTT, pH 7.2). After centrifugation (30 min., 12,000× *g*, 4 °C), the supernatant was rapidly used to determine the ACL activities by using the malate dehydrogenase coupled assay. The assay mixture contained 0.2 M Tris-HCl (pH 8.4), 10 mM MgCl_2,_ 20 mM trisodium citrate, 0.2 mM CoA, 10 mM ATP, 0.2 mM NADH, 0.4 U/mL MDH, and 10 mM DTT. The reaction was carried out at 37 °C for 30 min.; afterwards, the absorbance was measured through spectrophotometer at 340 nm. Blank was performed by omitting ATP or Coenzyme A. The activities of cyt-ACO were determined following the methods described before [43,44]. About 2.0 g sample was homogenized with 2 mL extraction buffer [0.2 mol/L Tris-HCL (pH 8.2), 0.6 mol/L sucrose, 10 mmol/L erythorbic acid], ice bath, and then ground with tissue grinder. After being centrifuged at 4 °C, 4000× g for 20 min., take the supernatant to volume to 5 mL, and then take 2 mL to centrifuge at 4 °C, 15,000× g for 15 min. Take the supernatant with extraction buffer [0.2 mol/L Tris-HCl (pH 8.2), 10 mmol/L erythorbic acid, 0.1% TritonX-100, and the volume was adjusted to 4 mL to obtain the cytoplasmic aconitase solution. Subsequently, the activities of cyt-ACO were determined according to the method that was described by Wang et al. [45]. The activities of ACCase and ACAT were determined by Enzyme-Linked Immunosorbent Assay (ELISA) Kits (plant) (Jiangsu Meibiao Biological Technology Co., Ltd., Jiangsu, China).

### 4.4. Vector Construction and Transformation

The open reading frame (ORF) sequences of *CitACLα1* and *CitACLβ1* [32] were amplified with the primers that are listed in Table S3 and cloned into the AatII/StuI and SpeI/XhoI sites of pK7WG2D vector to generate pK7-*ACLα1* and pK7-*ACLβ1*, respectively. The CaMV35S-*ACLβ1* terminator cassette was then inserted into the MluI/XhoI sites of pK7-*ACLα1* to create pK7-*ACLα1β1* overexpression vector. Table S4 lists the primers of the *CitACLβ1* RNA interference (RNAi) fragment. The fragment was cloned into pDONR221 and inserted into the RNAi vector pHELLS.gate.2. After confirming the vector sequences, constructs were transformed into the *Agrobacterium tumefaciens* strain GV3101 by the freeze-thaw method [46]. The generated constructs were subsequently transformed into citrus embryogenic callus according to the method described by Li, et al. [47]. Positive transgenic lines were screened according to the method of Cao, et al. [48] with little modification. Transgenic callus was selected on solid MT (Murashige and Tucker) basal medium containing 50 mg/L of hygromycin and 50 mg/L of kanamycin. Additionally, PCR amplification was then used to confirm the positive lines. Twenty-day-old callus was harvested for further experiments.

### 4.5. Quantitative Real-Time PCR (qRT-PCR) Analysis

The total RNA was extracted from citrus callus using a plant column RNA Extraction Kit (Sangon Biotech Company, Shanghai, China). First-strand cDNA was synthesized from 1 μg of total RNA while using a Prime Script RT Reagent Kit with a gDNA Eraser (TaKaRa, Dalian-China). qRT-PCR was performed with a Roche Light Cycler 480 Real-Time System (Roche, Switzerland) following the manufacturer’s protocol. The primers for qRT-PCR were designed by Primer 5.0 and are listed in Table S4. The reaction volume contained 5 μL SYBR Premix Ex Taq (TaKaRa, Dalian, China), 1 μL cDNA, 1 μM gene-specific primers, and 3 μL ddH2O. The qRT-PCR was conducted with three biological replicates, and each biological replication had three technical replicates. The reaction programs involved an initial incubation at 50 °C for 2 min, 95 °C for 10 min., and then followed by 40 cycles of 95 °C for 15 s, 60 °C for 15 s, and 72 °C for 20 s. The method of Livak, et al. [49] was employed to calculate the relative gene expression levels.

### 4.6. Statistical Analysis

Each determination was conducted with three biological replicates. The significance difference was evaluated by using Duncan’s multiple range test or *t*-test in the ANOVA program of SAS (SAS Institute, Cary, NC, USA). Moreover, the correlation was tested using the Pearson two-sided correlation coefficient of SPSS 17.0 software. Differences were considered to be significant at *p* < 0.05.

## 5. Conclusions

The variation of metabolites’ concentrations among citrus cultivars is a universal phenomenon, which might be regulated by a single factor or multiple factors. Although ACL and cyt-ACO have demonstrated their strategic role in the metabolite conversion, followed by ACCase and ACAT, they are not the determinants for the accumulation of related secondary metabolites in citrus fruits. However, ACL might have more influence on flavonoid concentration than carotenoid concentration in citrus fruits. 

## Figures and Tables

**Table 1:** Soluble sugar concentrations in fruit juice sacs during fruit development of six citrus cultivars.

Soluble Sugar	Cultivar	Sampling Time			
		T1	T2	T3	T4
Sucrose, mg·g^−1^ (FW)	Anliu	15.8 ± 0.45c	39.5 ± 1.61b	46.6 ± 0.99a	47.6 ± 2.53a
	Newhall	28.0 ± 1.22c	45.2 ± 1.27b	57.7 ± 1.32a	62.6 ± 2.78a
	Guoqing No. 1	16.4 ± 0.62d	35.5 ± 1.09c	56.9 ± 1.91b	64.3 ± 1.76a
	Huagan No. 2	11.2 ± 0.35c	12.1 ± 1.10c	20.0 ± 1.21b	40.4 ± 1.85a
	Zaoxiang	36.7 ± 1.99c	43.7 ± 4.63b	55.8 ± 3.21a	62.9 ± 4.05a
	HB	15.4 ± 1.17c	37.7 ± 1.16b	40.4 ± 2.25ab	45.8 ± 3.94a
Glucose, mg·g^−1^ (FW)	Anliu	15.0 ± 1.20d	18.3 ± 1.73c	25.2 ± 1.64b	39.9 ± 1.21a
	Newhall	9.0 ± 0.62d	12.6 ± 1.12c	36.9 ± 1.76b	41.8 ± 1.46a
	Guoqing No. 1	10.9 ± 1.46c	11.3 ± 1.19c	26.3 ± 2.35b	35.6 ± 2.71a
	Huagan No. 2	6.7 ± 0.58a	6.1 ± 0.72ab	5.6 ± 0.48b	4.7 ± 0.63b
	Zaoxiang	25.6 ± 1.12b	20.4 ± 1.03c	29.1 ± 1.34a	18.2 ± 1.06d
	HB	34.8 ± 2.21b	19.2 ± 1.56d	24.9 ± 1.41c	38.9 ± 1.59a
Fructose, mg·g^−1^ (FW)	Anliu	19.9 ± 1.31c	21.5 ± 1.15c	28.1 ± 1.53b	45.9 ± 1.52a
	Newhall	24.6 ± 1.11d	28.2 ± 1.28c	44.3 ± 2.84b	50.4 ± 1.83a
	Guoqing No. 1	14.7 ± 1.13c	15.6 ± 1.48c	34.7 ± 1.55b	44.5 ± 1.83a
	Huagan No. 2	5.5 ± 0.37a	5.3 ± 0.26a	4.8 ± 0.18b	3.6 ± 0.29c
	Zaoxiang	19.5 ± 1.53b	20.0 ± 1.24b	28.0 ± 2.87a	21.9 ± 1.51b
	HB	20.9 ± 1.46bc	17.2 ± 1.98c	23.4 ± 1.75b	40.2 ± 1.88a

Note: The exact sampling time of T1, T2, T3, and T4 in each cultivar was described in the Material and Method. Values are given as mean ± standard deviation (*n* = 3). Different lowercase letters between samples in the same row indicate significant difference at *p* < 0.05 by Duncan’s multiple range test.

**Table 2:** Organic acid concentrations in fruit juice sacs during the fruit development of six citrus cultivars.

Soluble Sugar	Cultivar	Sampling Time			
		T1	T2	T3	T4
Citric acid, mg·g^−1^ (FW)	Anliu	1.8 ± 0.15c	8.2 ± 0.18a	7.7 ± 0.15b	6.6 ± 0.20b
	Newhall	15.0 ± 0.49a	15.7 ± 0.45a	12.7 ± 0.32b	9.1 ± 0.19c
	Guoqing No. 1	16.5 ± 1.24b	21.0 ± 1.27a	9.8 ± 0.16c	9.1 ± 0.55c
	Huagan No. 2	28.7 ± 1.63a	16.6 ± 1.51b	10.8 ± 1.31c	5.0 ± 0.59d
	Zaoxiang	0.7 ± 0.10d	3.3 ± 0.12c	4.9 ± 0.14b	5.7 ± 0.38a
	HB	0.6 ± 0.11c	7.3 ± 0.18b	10.6 ± 0.48a	9.4 ± 0.92a
Malic acid, mg·g^−1^ (FW)	Anliu	1.3 ± 0.13ab	1.4 ± 0.08a	0.9 ± 0.04c	1.1 ± 0.14bc
	Newhall	2.0 ± 0.12a	0.6 ± 0.07b	0.5 ± 0.03b	0.6 ± 0.07b
	Guoqing No. 1	4.1 ± 0.25a	2.2 ± 0.04c	2.9 ± 0.18b	1.8 ± 0.16d
	Huagan No. 2	4.8 ± 0.22a	1.0 ± 0.06b	0.5 ± 0.06c	0.5 ± 0.02c
	Zaoxiang	0.4 ± 0.02a	0.4 ± 0.01a	0.1 ± 0.01c	0.3 ± 0.02b
	HB	0.7 ± 0.05a	0.5 ± 0.03b	0.1 ± 0.01c	0.1 ± 0.01c
Quinic acid, mg·g^−1^ (FW)	Anliu	2.7 ± 0.20a	1.6 ± 0.15b	1.0 ± 0.10c	0.6 ± 0.01d
	Newhall	2.3 ± 0.14a	1.2 ± 0.07b	0.5 ± 0.02c	0.3 ± 0.01d
	Guoqing No. 1	4.0 ± 0.10a	1.0 ± 0.06b	0.6 ± 0.02c	0.4 ± 0.01d
	Huagan No. 2	1.5 ± 0.09a	0.6 ± 0.01b	0.2 ± 0.01c	0.1 ± 0.00d
	Zaoxiang	1.6 ± 0.07a	0.6 ± 0.01b	0.4 ± 0.02c	0.0 ± 0.00d
	HB	2.7 ± 0.04a	0.5 ± 0.00b	0.5 ± 0.01b	0.0 ± 0.00c

Note: The exact sampling time of T1, T2, T3, and T4 in each cultivar was described in the Material and Method. Values are given as mean ± standard deviation (*n* = 3). Different lowercase letters between samples in the same row indicate a significant difference at *p* < 0.05 by Duncan’s multiple range test.

**Table 3:** Correlation coefficients between citrate concentration and ACL or cyt-ACO in each cultivar.

	Citrus cultivars					
	Anliu	Newhall	Guoqing No.1	Huagan No.2	Zaoxiang Pumelo	HB Pumelo
ACL	−0.8718	−0.6597	0.2552	0.1199	−0.0597	0.9583
cyt-ACO	−0.0275	−0.9224	−0.8170	−0.8953	0.8944	−0.4035

## Supplementary Materials

The following are available online at https://www.mdpi.com/2223-7747/9/3/350/s1, Table S1: Correlation coefficients between some parameters without considering cultivars. Table S2: Correlation coefficients between Acetyl-CoA concentration and the activity of ACCase or ACAT in each cultivar. Table S3: Primers for vector construction. Table S4: Primers for qRT-PCR. Figure S1: Expression of genes related to citrate biosynthesis and transport in transgenic citrus callus.
