# Supplementary material for: Comparative Metabolites and Citrate-Degrading Enzymes Activities in Citrus Fruits Reveal the Role of Balance between ACL and Cyt-ACO in Metabolite Conversions
Source: Plants (Basel). 2020 Mar 10;9(3):350. doi: 10.3390/plants9030350 (PMC7154853; doi:10.3390/plants9030350)
Supplement: Supplementary file 1 [file plants-09-00350-s001.pdf]

plants

MDPI

Supplementary Material

Table S1. Correlation coefficients between some parameters without considering cultivars.

	Citrate	ACL	Acetyl-CoA	ACCase	ACAT	Malonyl-CoA	Flavonoids	Carotenoids	cyt-ACO	GABA
**Citrate**	1	0.3946	0.216	0.099	−0.059	0.0183	0.3557	0.2559	−0.3404	0.2670
**ACL**		1	0.4333*	0.234	−0.0128	−0.1532	0.1412	−0.0314	−0.4431*	0.3218
**Acetyl-CoA**			1	0.2203	0.3497	−0.2549	0.4402*	−0.3349	−0.2446	0.3267
**ACCase**				1	−0.4324*	−0.2342	0.189	−0.08	0.1871	0.2329
**ACAT**					1	0.1969	0.1565	−0.2166	−0.1728	−0.0105
**Malonyl-CoA**						1	−0.0265	−0.0381	0.0687	0.0157
**Flavonoids**							1	−0.0341	−0.2504	0.2274
**Carotenoids**								1	−0.2125	0.2253
**cyt-ACO**									1	0.1959
**GABA**										1

*refers to the correlation coefficient is significant at $p < 0.05$.

Table S2. Correlation coefficients between Acetyl-CoA concentration and the activity of ACCase or ACAT in each cultivar.

	ACCase	ACAT
Anliu	0.1521	0.5840
Newhall	−0.6020	0.3797
Guoqing NO.1	0.2579	0.9711
Huagan NO.2	0.9746	0.9769
Zaoxiang pumelo	0.8251	−0.6857
HB pumelo	0.9457	0.1715

Table S3. Primers for vector construction.

Gene	Function	Forward Primer (5′-3′)	Reverse Primer (5′-3′)	Amplification Length
CitACLα1	Gene cloning	ATGGCTAGGAAGAAGATCAGAGAGTATG	CTATGATGCCGACATGATGCAGTCAATTG	1272 bp
CitACLβ1	Gene cloning	ATGGCTACAGGACAGCTGTTTTCAAAAAC	TTACTTGGTGTAGAGAACATCTTCCCATG	1827 bp
	Gene interference	TCACTGGATTTTGGTTTTAGGAA	TACCCGAATGGTTGGCTGT	316 bp

Table S4. Primers for qRT-PCR.

Gene	Forward Primer (5′-3′)	Reverse Primer (5′-3′)
PEPC1	GTGCGATCCCGTCTATCTGT	AAGGCTCAAGGCCACTTTTT
PEPC2	GGCATGCAAAACACTGGTTA	CATGTTCATTACGGCTTGGA
PEPC3	GAACAATGACGGACACAACG	TGGACTCGCTTCCAACTTCT
CS1	GGTGCCCCCAATATTAACAA	AGAGCTCGGTCCCATATCAA
PH8	CCGTGAAGGAATTGATTTGG	CCATGACAATGGATTCCACA

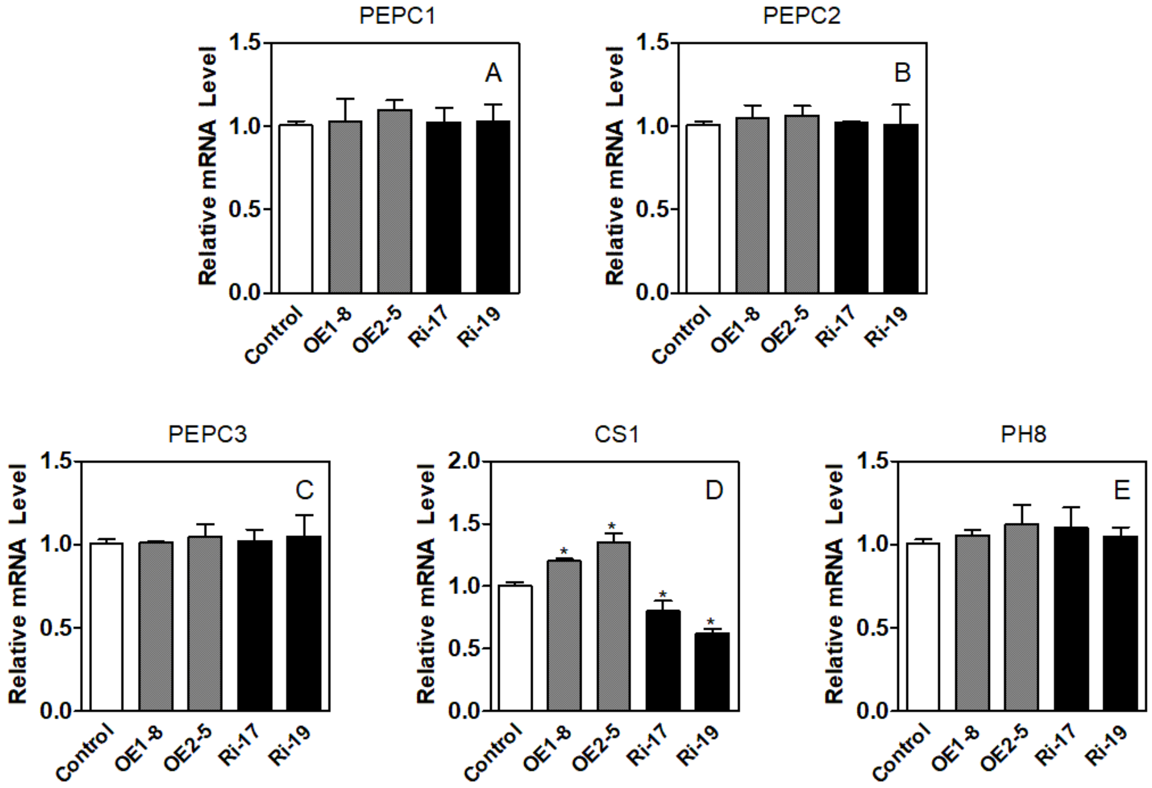

Figure S1. Expression of genes related to citrate biosynthesis and transport in transgenic citrus callus. PEPC refers to phosphoenolpyruvate carboxylase. CS refers to citrate synthase. PH8 refers to a P-type proton pump, *CsPH8* [18]. The primers used here were listed in Table S4. Asterisk (*) indicates that the difference is significant between the transgenic line and the control at $p < 0.05$ (t-test).
